# Zinc and Cadmium Mapping in the Apical Shoot and Hypocotyl Tissues of Radish by High-Resolution Secondary Ion Mass Spectrometry (NanoSIMS) after Short-Term Exposure to Metal Contamination

**DOI:** 10.3390/ijerph16030373

**Published:** 2019-01-29

**Authors:** Gabrijel Ondrasek, Peta L. Clode, Matt R. Kilburn, Paul Guagliardo, Davor Romić, Zed Rengel

**Affiliations:** 1 UWA School of Agriculture and Environment, The University of Western Australia, 35 Stirling Highway, Crawley WA 6009, Australia; zed.rengel@uwa.edu.au; 2 Faculty of Agriculture, The University of Zagreb, Svetosimunska cesta 25, 10 000 Zagreb, Croatia; dromic@agr.hr; 3 The Centre for Microscopy, Characterisation and Analysis, The University of Western Australia, 35 Stirling Highway, Crawley WA 6009, Australia; peta.clode@uwa.edu.au (P.L.C.); matt.kilburn@uwa.edu.au (M.R.K.); paul.guagliardo@uwa.edu.au (P.G.)

**Keywords:** Cd, Zn, ICP-MS, NanoSIMS, hypocotyl, shoot apex, food contamination

## Abstract

Zinc (as an essential phytonutrient) and cadmium (as a toxic but readily bioavailable nonessential metal for plants) share similar routes for crossing plant biomembranes, although with a substantially different potential for translocation into above-ground tissues. The in situ distribution of these metals in plant cells and tissues (particularly intensively-dividing and fast-growing areas) is poorly understood. In this study, 17-day-old radish (*Raphanus sativus* L.) plants grown in nutrient solution were subjected to short-term (24 h) equimolar contamination (2.2 µ*M* of each ^70^Zn and Cd) to investigate their accumulation and distribution in the shoot apex (leaf primordia) and edible fleshy hypocotyl tissues. After 24-h exposure, radish hypocotyl had similar concentration (in µg/g dry weight) of ^70^Zn (12.1 ± 1.1) and total Cd (12.9 ± 0.8), with relatively limited translocation of both metals to shoots (concentrations lower by 2.5-fold for ^70^Zn and 4.8-fold for Cd) as determined by inductively-coupled plasma mass spectrometry (ICP-MS). The in situ Zn/Cd distribution maps created by high-resolution secondary ion mass spectrometry (NanoSIMS, Cameca, Gennevilliers, France) imaging corresponded well with the ICP-MS data, confirming a similar pattern and uniform distribution of ^70^Zn and Cd across the examined areas. Both applied techniques can be powerful tools for quantification (ICP-MS) and localisation and visualisation (NanoSIMS) of some ultra-trace isotopes in the intensively-dividing cells and fast-growing tissues of non-metalophytes even after short-term metal exposure. The results emphasise the importance of the quality of (agro)ecosystem resources (growing media, metal-contaminated soils/waters) in the public health risk, given that, even under low contamination and short-term exposure, some of the most toxic metallic ions (e.g., Cd) can relatively rapidly enter the human food chain.

## 1. Introduction

Understanding of trace metal homeostasis and phyto-physiological processes has expanded considerably in recent decades and is being used in phytoremediation technologies (i.e., clean-up of metal-contaminated land by plants) (e.g., [1]), bio-fortification of food (i.e., improving nutritive value of crops regarding essential trace metals) (e.g., [2]) as well food safety/security from metal contamination (i.e., growing “metal-clean” food) [3]. Given that trace metals can accumulate in edible tissues (particularly underground ones, such as roots, tubers and hypocotyls) and/or primary nutrient deposition sites (e.g., young shots), consumption of vegetables (especially fresh (non-processed) that are nutritive highly valued) is one of the main entry routes of certain trace metals (Cd, Zn, Hg) into humans [4,5,6,7]. Over the last several decades, trace heavy metal Cd was one of the most frequently reported hazards (after mycotoxins, pathogens and pesticide residues) in the Rapid Alert System for Food and Feed [7] regulated by the European Food Safety Authority. In addition, Cd is recognised as one of the high-priority contaminants/carcinogenics by other national and international authorities such as the Environmental Protection Agency in the US or the International Agency for Research on Cancer [5]. 

In most organisms (except, e.g., marine phytoplankton [8,9]), Cd has no biological function, representing threat to environmental/public health due to its role in a wide range of toxicological (e.g., growth retardation, yield reduction, and necrosis) and physiological disorders (e.g., hormonal disruption, carcinogenesis, alterations in vitamin metabolism and (re)absorption of nutrients, triggering secondary (oxidative) stress) [4,6,10] (see also review by Huang et al. [5]). It is well known that similar physicochemical properties of the bioavailable forms of zinc (as an essential micronutrient; Zn^2+^) and cadmium (Cd^2+^) (e.g., [9]) result in competition for adsorption sites in the rhizosphere, uptake across the root–cell plasma membrane, and transport to shoots [4]. Thus, increased relative abundance of one element (e.g., Zn) could suppress the rhizosphere-to-plant transfer and deposition of the other (e.g., Cd). In higher plants, vascular tissues (i.e., xylem and phloem) are crucial for transport, distribution and deposition of essential and non-essential trace metal elements (e.g., [11]) from roots to shoots [12]. 

In hypocotyls (i.e., specific anatomic structure representing lower part of stem and upper part of root), cells grow radially from the vascular cambium, undergoing gradual differentiation into either xylem parenchyma and vessels (centrally) or phloem (peripherally) (e.g., [13]). In some widely grown vegetables such as radish (*Raphanus sativus* L.), pronounced cambial activity results in a thickened (fleshy/well hydrated) hypocotyl, with xylem area representing a substantial part of radish hypocotyl cross section [14], allowing for high flow of water and dissolved substances and thus a high potential for accumulation of nutrients, proteins, carbohydrates, fibres, and vitamins [15,16] as well as potential contaminants such as Cd [17]. 

Shoot apex comprises mostly apical meristem and leaf primordia, representing one of the fastest growing plant tissues requiring intensive inflow of water and nutrients [18]. Accordingly, shoot apex quickly perceives any potential nutritional changes (imbalances) and/or the presence of contaminants. Hence, hypocotyl and shoot apex tissues are suitable for early detection and visualisation of changes in the supply of metallic micronutrients (e.g., Zn) as well as contaminants (e.g., Cd). 

High resolution secondary ion mass spectrometry (NanoSIMS) is a powerful and sensitive in situ mapping technique for the visualisation of ultra-trace elements in biological samples, potentially able to ensure specific compositional information at a scale of 50 nm to few microns [19,20]. Microscopy incorporating SIMS technique is based on bombarding a specially-prepared sample with a high-energy primary ion beam (Cs^+^, O^−^) that sputters atoms, molecules and electrons (i.e., ionised species or secondary ions) from the sample surface, to be separated on the basis of their mass-to-charge ratio using a high-performance mass detector [21,22].

However, the major limitations of the NanoSIMS are: (i) relatively low sensitivity in detection of some elements (e.g., Zn and Cd) due to poor generation of secondary ions after bombardment by the primary beam; (ii) the difficulty of quantifying actual metal concentrations across the scanned area; (iii) the inability to obtain chemical speciation (state) of metals; and (iv) a need to balance preservation of the natural structure in biological specimens against sample preparation requirements for SIMS measurements (e.g., obtaining as flat as possible a scanned area surface is difficult given high water content) [20,23]. Thus, mapping of Cd and Zn at the nano-scale in metal-sensitive (yet edible) plant parts and/or in tissues with low metal concentration (e.g., shoots) might be additionally challenging because of potentially low signal (i.e., metal concentration down to several mg/kg). To overcome these difficulties, researchers used relatively long-term studies (e.g., few months) and/or unusually high concentrations of metals (e.g., [1,24,25]) that might cause phytotoxicity, therefore diminishing the environmental and physiological relevance of results obtained. 

In our previous study [23], we confirmed that epidermal cells in the root apex of metal-sensitive radish plants can dominantly accumulate Cd and ^70^Zn after 24-h exposure, suggesting relatively weak root–hypocotyl–shoot translocation and deposition. To test that hypothesis, the main objectives in the present study were to use short-term (24 h) exposure of metal-sensitive radish plants to equimolar (2.2 µ*M*) concentration of cadmium and zinc in the rhizosphere to examine the in situ Zn/Cd distribution in the xylem area of hypocotyl (organ widely consumed by humans) as well as fast-growing shoot apices using NanoSIMS.

## 2. Experimental Set-Up and Methodology 

### 2.1. Plant Growth Conditions

Radish (*Raphanus sativus* L. cv. Cherry Belle) was cultivated in a fully-controlled growth chamber (12/12 h light/dark period, 350 µM m^−2^ s^−1^ photosynthetically active radiation supplied by high-pressure metal-halide lamps, air temperature 22/17 °C, and air humidity 60/80%) at the University of Western Australia as described previously [23]. In short, uniform seeds of radish were surface-sterilised by soaking in 70% (v/v) ethanol for 1 min followed by 1% (v/v) sodium hypochlorite for 5 min, and germinated in ultrapure deionised water (18 mΩ cm^−1^) obtained from a Milli-Q system (Millipore Corp, Milford, CT, USA) for next 48 h. Six uniformly germinated seeds were then transferred to the floating plastic net in 4 L pots filled with half-strength nutrient solution (see below). Six days after germination, three uniform seedlings, supported by 2-cm-long and 2-cm-wide poly-foam pipe were positioned in holes cut into plastic lids of 4 L pots filled with full-strength nutrient solution containing (in mM) Ca(NO_3_)_2_ 2.5, KNO_3_ 2.5, KH_2_PO_4_ 0.5, MgSO_4_ 1.0, MES 1 m*M* (at pH 6.0), and (in µM) FeSO_4_ 50, H_3_BO_3_ 5.0, MnCl_2_ 3.70, ZnSO_4_ 0.64, CuSO_4_ 0.52, NiSO_4_ 0.1 and Na_2_MoO_4_ 0.02 (Figure 3A). Nutrient solutions were continuously aerated and changed every 48 h. The Milli-Q ultrapure water was used during the whole experiment, including preparation of stock/nutrient solutions, standard/blank solutions and plant samples. All chemicals used were of analytical grade.

On 17th day after germination, the full-strength nutrient solution was supplemented with equimolar (2.2 µ*M*) concentration of Cd (as cadmium nitrate tetrahydrate, Ajax Chemicals Ltd., Sydney, Australia) and ^70^Zn (as zinc sulphate heptahydrate containing 95.42% ^70^Zn, Trace Sciences International Corp., Ontario, Canada) in triplicate. To enhance metal desorption from roots before imposing the ^70^Zn/Cd treatments, the roots of test plants were firstly immersed into Milli-Q water for 5 min, transferred to 5 m*M* CaCl_2_ for 10 min and again to Milli-Q water for 15 min. After 24-h exposure to the ^70^Zn/Cd treatments, different radish tissues were sampled (Figure 3A). 

### 2.2. Sampling and Sample Preparation for the NanoSIMS 

Each radish plant was separated by a scalpel blade into four main parts: roots, hypocotyl, shoot and shoot apex (a few millimetres long) (Figure 3A). The hypocotyl and shoot apex were used in this study by cutting several sections (approximately 2–5 mm in length and 1 mm in diameter) from each in each of three replicates, and immediately immersing them in liquid N slush to fix and preserve metallic isotopes of interest. The hypocotyl and shoot sections were then stored in liquid N until the process of freeze substitution. Freeze substitution of liquid N-stored sections was performed in an anhydrous mixture of 10% acrolein and diethyl ether over 24 days using a fully-controlled freeze-substitution system (Reichert AFS, Leica) according to the published procedures (for more details, see [26]). After 24 days, sampled sections were washed in acrolein (thrice for 20 min each time) and then embedded gradually in Araldite 502 resin, using graded resin–acrolein (v/v) mixtures of 10%, 25%, 50% and 75% for 2 h each and then 100% resin overnight. Over the next 4 days, the samples were kept in 100% Araldite 502 resin with changes twice daily. Individual sampled sections were finally placed into moulds filled with resin and polymerised at 60 °C for 24 h under vacuum.

One-micrometre-thick sections of the resin-embedded radish fruit and shoot apex samples were cut by glass knife on an ultramicrotome (Leica), and then transferred onto glass slides as described previously [27], stained with 0.2% (v/v) toluidine blue (Sigma-Aldrich) and observed under a light microscope (Olympus CH). Identified sections with particular areas of interest were imaged by Zeiss Axioskop 2 plus, and then processed in software AxioVision ver. 3.1. After identification of particular sections (e.g., well-developed and preserved cell structures of xylem tissue in the hypocotyl and mesophyll cells in leaf primordia), a few new sections of radish material were cut and placed onto silicon wafers (7 mm × 7 mm), coated with a 10 nm layer of gold and loaded into the NanoSIMS vacuum chamber.

### 2.3. NanoSIMS Detection

In situ chemical maps of five isotopes were simultaneously acquired with high resolution (down to 50 nm) using the NanoSIMS 50 (Cameca, Gennevilliers, France) at the Centre for Microscopy, Characterisation and Analysis, University of Western Australia. All areas of interest (approximately 50 µm × 50 µm) were firstly pre-sputtered with the primary ion beam to remove surface contamination and enhance the generation of secondary ions [28]; after that, positive secondary ions were sputtered using an O^−^ primary beam with the same set-up as was used in our study of root apices (beam current 30 pA, beam size approximately 300 nm, and impact energy approximately 16 keV). The instrument was tuned to high mass resolution (6000 mass resolving power) to minimise/exclude isobaric interferences. Ion images were acquired at a raster size of 50 µm^2^, with a resolution of 256 × 256 pixels and counting times of 100 ms per pixel. In addition, high-resolution mass spectra were acquired from the standards and compared with the signal from the samples to avoid peak overlaps. The numerical data were extracted directly from the ion images by selecting regions of interest and pixels defining certain structural features on the normalised and composite images using the OpenMIMS plugin for ImageJ (https://nano.bwh.harvard.edu/openmims, Harvard, Cambridge, MA, USA; [29]).

### 2.4. Sampling and Sample Preparation for ICP-MS Analysis 

After sampling for the NanoSIMS, the remaining hypocotyl and radish shoot (all leaf material and the rest of shoot apex) from each replicate was dried (at 70 °C for 48 h) and digested in nitric (HNO_3_) and perchloric acids (HClO_4_) according to the procedure explained by Ondrasek et al. [17]. Briefly, the hypocotyl and shoot samples were separately weighed into a 50 mL conical flask and digested firstly in 5 mL of concentrated HNO_3_ at 95–100 °C (~50 min for hypocotyls and ~40 min for shoots) until all plant material was dissolved and brownish fumes subsided. The digests were allowed to cool down to room temperature and were again digested with 0.5 mL of concentrated HClO_4_ and heated to 145–150 °C (~30 min) until the flasks filled with thick, white fumes. After cooling, the digests were transferred with three rinses by ultrapure water to 10 mL vials containing 5 µg of yttrium serving as an internal standard (to account for instrument drift and sensitivity). In each analytical batch, at least three reagent blanks with the ultrapure water, and one internationally certified reference plant material (lucerne 159, WEPAL, Wageningen, The Netherlands) were included. The total concentration of ^70^Zn, Zn and Cd in digests was determined using an Inductively-Coupled Plasma Mass Spectroscopy (ICP-MS) instrument (Agilent Technologies 7700x, Santa Clara, CA, USA). The detected concentrations of all reference standard samples and blanks yielded results that were within declared values (i.e., differing by <5%). The ICP-MS data were statistically analysed using the SAS software package ver. 9.3 (SAS Institute Inc. NC, USA).

## 3. Result and Discussion

### 3.1. Total Concentration of Zn and Cd

**Figure 1:**
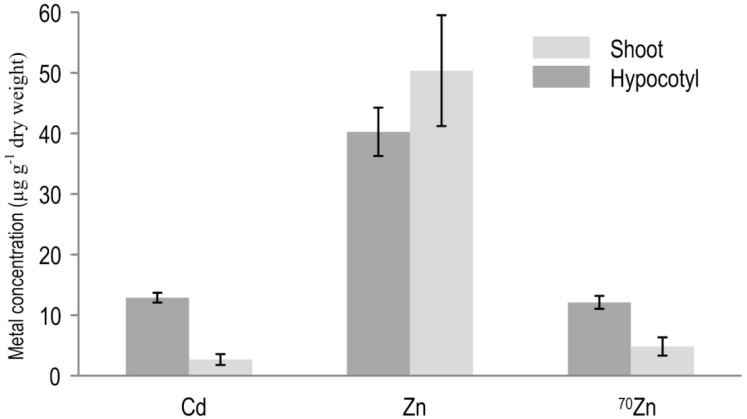
ICP-MS measured concentration of total Cd, total Zn and ^70^Zn in the bulk hypocotyl and shoot tissue of 18-day-old radish (*Raphanus sativus* L. cv. Cherry Belle) after 24-h exposure of root system to the equimolar (2.2 µ*M*) Cd/^70^Zn treatment. Error bars represent ± SE (n = 3).

Before sample preparation for the NanoSIMS data acquisition, ICP-MS analyses of the total hypocotyl and shoot tissue of 18-day-old radish plant were performed to check their appropriateness for in situ mapping, given the sensitivity limit of the NanoSIMS procedures. ICP-MS is very often employed as a (pre)screening method prior to isotopic mapping by NanoSIMS (e.g., [24]) given restricted sensitivity of SIMS analysis [23,27]. Namely, SIMS is sensitive down to mg/kg (in appropriate sample/analyte combinations) and isotopic variations of elements from ^1^H to ^238^U can be detected (e.g., [30]), while ICP-MS as one of the routinely used techniques for detection of metal isotopes, enables even higher sensitivity (down to µg/kg) in a wide range of biological matrices (e.g., [31]). Both metals of interest, ^70^Zn (natural abundance < 1%) and Cd, were used at 2.2 µ*M* each in the nutrient solution. ICP-MS analyses showed similar concentrations of both metals in radish hypocotyl (Cd 12.9 ± 0.8 and ^70^Zn 12.1 ± 1.1 µg/g) as well in the radish shoot (Cd 2.7 ± 0.9 and ^70^Zn 4.8 ± 1.5 µg/g) (Figure 1). It was confirmed that both metals were poorly transported from the taproot and lateral roots, with total accumulation markedly reduced in edible hypocotyl (Cd by 27-fold, ^70^Zn by 15-fold) and shoots (Cd by 132-fold, ^70^Zn by 38-fold) in comparison to roots (cf. [23] and Figure 1). However, results clearly show that, even under short-term exposure to relatively low concentrations, micronutrient Zn^70^ and toxic ^114^Cd were easily and rapidly translocated from the rhizosphere solution into the edible radish hypocotyl and shoot tissues at a similar rate. Such results are in a line with the most recent review by Khalid et al. [32] who noted that using wastewater (contaminated with metals and other pollutants) for irrigation purposes markedly increased concentration of toxic elements (Cd, Ni, Cr) in vegetable and cereal crops (by at least two-fold in comparison with crops irrigated by non-contaminated water). For example, irrigation with sewage water (vs. non-contaminated water) increased Cd concentration in radish hypocotyl about eight-fold (from around 2 to 16.2 mg/kg) and Zn almost four-fold (from around 36 to 137 mg/kg) [33]. In addition, growing radish in Cd-contaminated (5 mg Cd/kg) organic soil (vs. non-contaminated soil with Cd background concentration of 0.3 mg/kg) increased Cd concentration in radish shoots 20-fold (from 3 to 60 mg/kg) and in edible hypocotyl 16-fold (from 0.7 to >11 mg/kg) [34]. These Cd shoot concentrations exceeded the toxic threshold (~10 mg/kg d.w.) for non-methalophytes (e.g., [23,35]).

**Figure 2:**
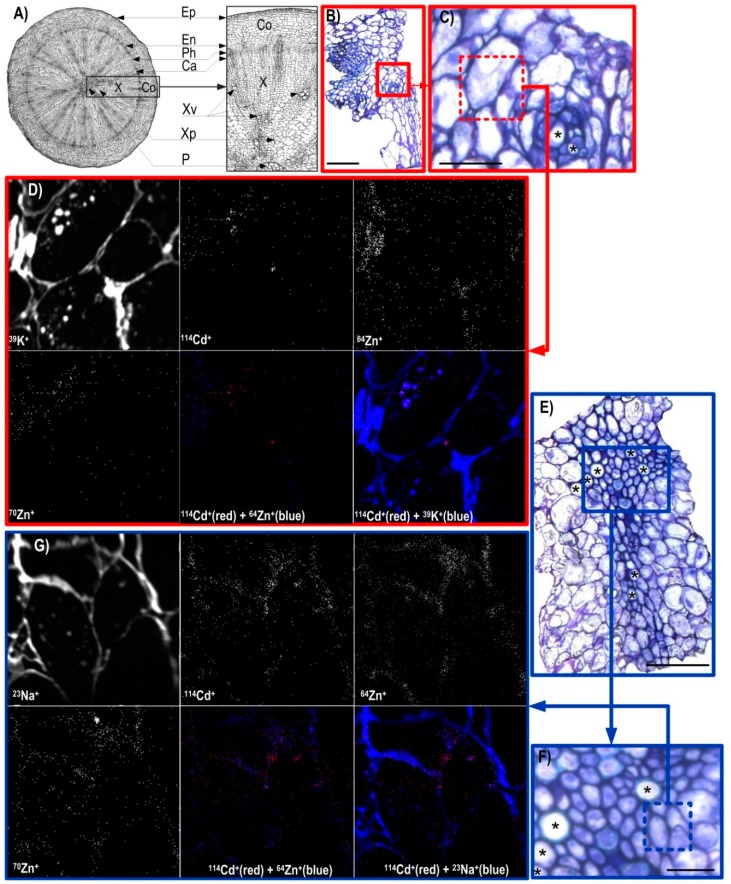
(**A**) Schematic presentation of fully-developed radish hypocotyl tissue in the cross section (left) with a part enlarged (right). Ep, epidermis; Co, cortex; En, endodermis; Ph, phloem; Ca, cambium; X, xylem; Xv, xylem vessel; Xp, xylem parenchyma; P, Pith. (**B**,**C**,**E**,**F**) Optical micrographs of the Araldite 502 resin-embedded transversal sections of 18-day-old radish (*Raphanus sativus* L. cv. Cherry Belle) edible hypocotyl tissue exposed to the equimolar (2.2 µ*M*) Cd/^70^Zn treatment for 24 h and prepared by freezing in liquid N slush followed by freeze substitution and staining with toluidine blue. Sections were sampled from the xylem area of hypocotyl. In (**C**,**F**), dotted squares represent the regions of interest (shown in (**D**,**G**), respectively) scanned by the NanoSIMS, and asterisks indicate xylem vessels. Bars represent 100 µm (**B**,**E**) and 50 µm (**C**,**F**). In (**D**,**G**), the high-resolution secondary ion mass spectrometry (NanoSIMS) images (50 µm × 50 µm) were obtained using the O^−^ primary ion beam and processed by multi-isotope imaging mass spectrometry (MIMS) to indicate the distribution of ^23^Na^+^, ^39^K^+^, ^64^Zn^+^, ^70^Zn^+^ and ^114^Cd^+^ as separate and composite (dual) image.

Radish is widely cultivated non-methalophyte, with a short vegetation period and adaptable to various (agro)ecological conditions. It is mostly consumed as non-processed, fresh foodstuff what helps preserve its full nutritional value, making radish a rich source of nutritive, medical and pharmaceutical components such as nutrients, carbohydrates, proteins, crude fibres, folic acid, ascorbic acid, sulforaphane, peroxidase, isothiocyanates, etc. [15,16,36]. As shown in Figure 2A, the hypocotyls had an outer epidermal layer with relatively small cells bordering the cortex layer. Although the epidermal layer of hypocotyl can take up water and dissolved nutrients when grown in soil, in this study the hypocotyls were positioned above the metal-containing solution with no contact with it, ensuring that all nutrients and contaminants found in the hypocotyl would have been delivered there via xylem after uptake by roots (Figure 2A). No hypocotyl formed lateral roots/hairs (data not shown), although that is possible in soil culture and/or nutrient solution conditions (e.g., [17,34]). 

Xylem comprised vessels and accompanied parenchyma cells, positioned inward of the cambial zone (Figure 2A); xylem represented the majority of the fast-growing hypocotyl tissues, especially during hypocotyl secondary growth (thickening). Hypocotyl is edible storage radish organ, whose thickening determines final yield and quality [16]. Hypocotyl thickening in radish occurs through primary (cortex splitting) and secondary stages (expansion growth) (e.g., [16]). The hypocotyl thickening process is dependent on accelerated activity of vascular cambium tissue and formation of secondary xylem (and notably parenchyma cells) and phloem (Figure 2) [15]; hypocotyl thickening enlarges a potential sink for metals. In the study presented here, Zn and Cd were applied during the expanding stage of hypocotyl thickening, i.e. during intensive growth, when upward vascular transport is strong. In addition, during that stage, the biosynthesis, metabolism and accumulation of organic compounds are intensive [15,16]; these compounds are crucial in organo-complexation of metallic ions (e.g., [17,37]). Relatively large xylem vessel cells (rectangular-to-rounded in shape; Figure 2C,E,F) are mainly responsible for upward (root–hypocotyl–shoot) transport of water and dissolved elements [38]. Accordingly, we expected that the area in the vicinity of xylem vessels would be enriched in metals of interest (Cd/Zn); thus, these areas were chosen for examination by the NanoSIMS.

**Figure 3:**
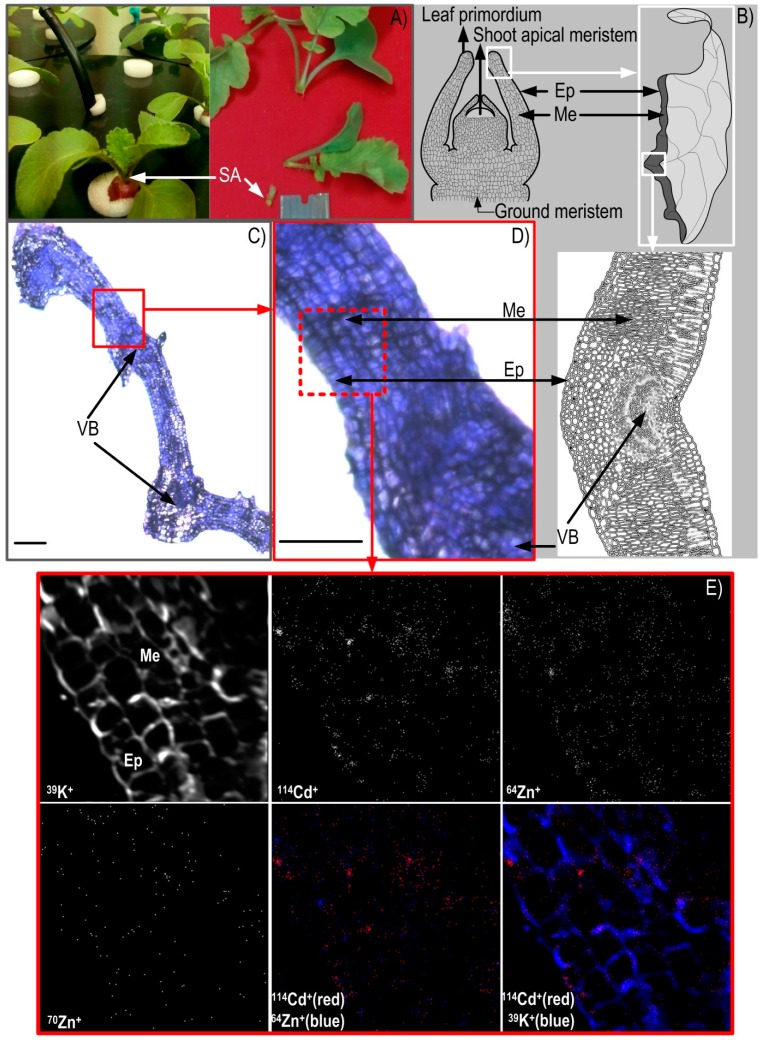
(**A**) Cultivated 18-day-old radish plants (*Raphanus sativus* L. cv. Cherry Belle) after 24-h exposure to the equimolar (2.2 µ*M*) Cd/^70^Zn treatment and a shoot apex (SA) during sampling. (**B**) Schematic presentation of vertical section of the shoot apex (left) and details of leaf primordia (right, bottom) tissue. Ep, leaf epidermis; Me, leaf mesophyll, VB, vascular bundle. (**C**,**D**) Optical micrographs of the Araldite 502 resin-embedded transverse sections of radish leaf primordia exposed to the equimolar (2.2 µ*M*) Cd/^70^Zn treatment for 24 h and prepared by freezing in liquid N slush followed by freeze substitution and staining with toluidine blue. On the magnified area (**D**), the dotted square represents the region of interest scanned by the NanoSIMS. Bars represent 100 µm (**C**) and 50 µm (**D**). (**E**) The high-resolution secondary ion mass spectrometry (NanoSIMS) images (50 µm × 50 µm) obtained using the O^−^ primary ion beam and processed by multi-isotope imaging mass spectrometry (MIMS) to indicate distribution of ^39^K^+^, ^64^Zn^+^, ^70^Zn^+^ and ^114^Cd^+^ as separate and composite (dual) image.

The radish shoot apex comprised poorly differentiated meristematic cells and leaf primordia (Figure 3A,B). The shoot apex is one of the fastest growing aboveground regions, thus requiring an intensive supply of water and nutrients [18] that arrive predominantly via the xylem. Consequently, the shoot apex would be expected to accumulate nutrients and contaminants applied as a short-term pulse, and was therefore characterised in situ by the NanoSIMS. 

### 3.2. NanoSIMS Maps of Metals of Interest in the Hypocotyl and Shoot Apex

A combination of low ionisation and high solubility (mobility) of Zn and Cd makes them some of the most challenging trace elements for in situ detection and visualisation in biological samples by the NanoSIMS technique [20,23]. Indeed, crucial for the NanoSIMS mapping is to keep as faithfully as possible the in vivo spatial distribution of soluble/mobile elements during specimen preparation without disturbing hydrated and fragile structures in the fast-growing aboveground (shoot apex) and belowground (hypocotyl) radish tissues. With that aim and prior to ultimate specimen embedding in Araldite 502 resin, tissue sections that had been snap-frozen in liquid N were gradually freeze-substituted in an acrolein/diethyl ether mixture as one of the best techniques for preservation of (sub)cellular structures for maintaining elemental distributions in plant specimens for NanoSIMS studies [22,23,24,27]. 

Optical micrographs of Araldite 502 resin-embedded, transversal, 1-µm thick sections of radish hypocotyl (Figure 2B,C,E,F) as well as shoot apex (leaf primordia) (Figure 3C,D) showed well-developed and structurally-preserved cells, albeit with occasional spots of mechanical damage outside the areas examined by the NanoSIMS. Figure 2B and its magnified section 1 (Figure 2C), as well as Figure 2E and its magnified section 2 (Figure 2F), clearly show xylem vessels embedded in the surrounding parenchyma tissue. In the shoot apex, it was possible to distinguish the outer and inner epidermis bordering the mesophyll cells and vascular bundles (Figure 3C,D). 

The CAMECA NanoSIMS 50 is a dynamic SIMS instrument and enables the simultaneous collection of five isotopes, with high mass and spatial resolution. A primary O^−^ ion beam was scanned across the surface of the sections (50 µm × 50 µm), and the sputtered secondary ions were extracted to a double-focusing mass spectrometer ensuring acquisition of positive secondary ion elemental maps of the isotopes of interest (^64^Zn, ^70^Zn and ^114^Cd) as well as ^23^Na and ^39^K. The ^39^K^+^ and ^23^Na^+^ images confirmed the outline and the structural integrity of the cells in the xylem area of the hypocotyl and at the epidermal-mesophyll boundary in leaf primordia (Figure 2D,E,G). Due to a relatively high positive secondary ion yield [39], chemical maps of ^23^Na^+^ and ^39^K^+^ across the scanned sections were brighter and clearer in comparison to other isotopic images (Figure 2D,G and Figure 3E). In this study, the maps of ^23^Na^+^ and ^39^K^+^ displayed their strong enrichment along the lines that corresponded with the cell wall structures in either the hypocotyl or shot apex (Figure 2D,G and Figure 3E), as well as around intracellular granulate structures in the hypocotyl alone (Figure 2D,G). The successful NanoSIMS in situ imaging of ^23^Na^+^ and ^39^K^+^ as some of the most soluble ions confirmed that the specimen preparation method (i.e., gradual freeze substitution of liquid N-stored specimens) was successful, with negligible redistribution of observed ions (e.g., [27]) and good preservation of the in vivo situation. This specimen preparation technique was also shown to be successful in the previous NanoSIMS studies of (sub)cellular localisation of different mobile elements such as Na, K, Mg, Ni and others in *Alyssum lesbiacum* (Candargy) Rech.f. (*Brassicaceae*) [22] and Zn (as well as As, Cu, Fe, Mn, etc) in rice tissues [24,27]. 

Zinc as a nutrient and Cd as a phytotoxic trace element are highly mobile in hydrated organic environments such as plant (hypocotyl/shoot) tissues, but, contrary to Na and K, Zn and Cd generate substantially lower secondary ion yields after bombardment by an O^−^ beam in NanoSIMS, making Zn and Cd highly challenging elements for in situ mapping by the NanoSIMS in plant samples [20,23]. In the present study, the Zn and Cd signals were weaker than those of Na and K (Figure 2D,G and Figure 3E). However, given the longer exposure of plants (since germination) to ^64^Zn compared to ^70^Zn and Cd, its signal (and concentration) was higher and maps in the examined areas were clearer, although precise (sub)cellular localisation remained elusive (Figure 2D,G and Figure 3E). A similar pattern, showing a uniform distribution of trace metal isotopes (^70^Zn^+^ and ^114^Cd^+^) was seen in both the leaf primordia (epidermis-mesophyll) and the hypocotyl (area around xylem vessels). In the case of hypocotyl xylem sections (Figure 2D,G), maps showed co-localisation of ^64^Zn^+^, ^70^Zn^+^ and ^114^Cd^+^, whereas in leaf primordia (Figure 3E) co-localisation between ^64^Zn^+^ and ^114^Cd^+^ was detected. These observations suggest that Cd and Zn isotopes not only follow similar pathways in the root–hypocotyl–shoot continuum (e.g., [23,40,41]), but have similar distribution in intensively-growing tissues as well. 

From the perspective of public health risk from metal contamination/influence, the presented results (Figure 1, Figure 2D,G and Figure 3E) clearly highlight the importance of the quality of natural resources, notably cultivated land areas and water resources (e.g., metal-contaminated agricultural soils or waters used for irrigation) in producing “clean” and nutritious (healthy) food with balanced contents of essential/beneficial nutrients. Our results confirm that, even under short-term metal exposure and naturally relevant low contamination (e.g., [5]), Cd as one of the most toxic metals easily and rapidly entered edible crop tissues and thus human food chain, potentially compromising our health.

Finally, large variation exists among plant species and genotypes in accumulation of trace metals in roots (hypocotyl) and distribution to shoots [4,5,40]. Significant research effort with different radish genotypes has been devoted to elucidation of trace element pathways from rhizosphere to plants [36] as well to their morphological/anatomical [42], physiological [43], and biochemical and genomic [15,16,44,45] characterisation. The study presented here, in conjunction with some recent observations [23], clearly showed that uptake, transport and deposition of Cd and Zn are similar in widely-consumed radish hypocotyls and shoots. Future research may explore potential differences among radish genotypes to accumulate Cd as one of sustainable approaches for utilising land with elevated Cd concentration in soil [15] and lowering the risk of toxic Cd entering into our food chain. Traditional breeding techniques in combination with transgenic approaches have been highlighted as a promising strategy for improving crop food/feed production in certain environmentally constrained conditions such as elevated Cd concentration in soil [4,5,15] and increased salinity [46]. For instance, Yu et al. [47] identified 30 rice cultivars (among 43 tested) that maintained Cd concentration in grain within the limits safe for human consumption when grown on Cd-contaminated soil (1.75 mg Cd/kg soil). Some spinach genotypes have the capacity to retain Cd in the root cell wall, thus decreasing root-to-shoot Cd translocation [48]. However, selection and usage of genotypes with a decreased potential for Cd uptake and accumulation should be considered carefully because low Cd uptake may mean low uptake of chemically similar essential nutrients (e.g., Zn, Cu, and Ca), compromising the nutritional quality of edible plant parts. 

## 4. Conclusions

To the best of our knowledge, this is the first report of detailed localisation of low concentrations of ^114^Cd and ^64^Zn/^70^Zn in the shoot apex (leaf primordia) and hypocotyl of radish (non-metalophyte species) exposed to relatively low concentrations of metallic ions over a short period of time. Precise quantification (ICP-MS) as well localisation and visualisation (NanoSIMS) of trace metals in rapidly-dividing and differentiating cell structures of metal-sensitive radish proved to be a powerful approach. The tissue concentrations of ^70^Zn were about 2.5-fold lower and of Cd 4.8-fold lower in the shoots than the hypocotyl. NanoSIMS maps of the apical shoot (epidermis–mesophyll boundary) and hypocotyl tissues (around xylem vessels) showed a similar pattern and uniform distribution of ^70^Zn and ^114^Cd. These observations suggest that Zn and Cd follow similar pathways along the root–hypocotyl–shoot continuum in radish. Although the study was performed in nutrient solution, the results highlight the importance of the quality of soil and water in food production on the human health risk, with Cd entering the edible tissues even after short-term exposure to low Cd contamination, potentially endangering the public health. Although NanoSIMS has relatively high spatial resolution sensitivity (e.g., about 300 nm with an O^−^ probe) and mass resolution for isotopic mapping at (sub)cellular scales, the mapping of low concentrations of Zn and Cd isotopes at a nanoscale remains a technically difficult task in non-metallophytes.
